# Improving the care of people with traumatic brain injury through the Neurotrauma Evidence Translation (NET) program: protocol for a program of research

**DOI:** 10.1186/1748-5908-7-74

**Published:** 2012-08-07

**Authors:** Sally E Green, Marije Bosch, Joanne E McKenzie, Denise A O’Connor, Emma J Tavender, Peter Bragge, Marisa Chau, Veronica Pitt, Jeffrey V Rosenfeld, Russell L Gruen

**Affiliations:** 1 School of Public Health and Preventive Medicine, Monash University, Melbourne, Australia; 2 Department of Surgery, Monash University / National Trauma Research Institute, Melbourne, Australia; 3 Department of Surgery, Monash University / Department of Neurosurgery, The Alfred Hospital / National Trauma Research Institute, Melbourne, Australia; 4 Department of Trauma, The Alfred Hospital / Department of Surgery, Monash University / National Trauma Research Institute, Melbourne, Australia

**Keywords:** Knowledge translation research, Study protocol, Neurotrauma, Traumatic brain injury

## Abstract

The Neurotrauma Evidence Translation (NET) program was funded in 2009 to increase the uptake of research evidence in the clinical care of patients who have sustained traumatic brain injury. This paper reports the rationale and plan for this five-year knowledge translation research program. The overarching aims of the program are threefold: to improve outcomes for people with traumatic brain injury; to create a network of neurotrauma clinicians and researchers with expertise in knowledge translation and evidence-based practice; and to contribute knowledge to the field of knowledge translation research. The program comprises a series of interlinked projects spanning varying clinical environments and disciplines relevant to neurotrauma, anchored within four themes representing core knowledge translation activities: reviewing research evidence; understanding practice; developing and testing interventions for practice change; and building capacity for knowledge translation in neurotrauma. The program uses a range of different methods and study designs, including: an evidence fellowship program; conduct of and training in systematic reviews; mixed method study designs to describe and understand factors that influence current practices (*e.g.*, semi-structured interviews and surveys); theory-based methods to develop targeted interventions aiming to change practice; a cluster randomised trial to test the effectiveness of a targeted theory-informed intervention; stakeholder involvement activities; and knowledge translation events such as consensus conferences.

## Background

Traumatic Brain Injury (TBI) is an important global health problem. It is defined as injuries caused by external mechanical force to the head, *e.g.*, in motor vehicle accidents, falls, sporting accidents, violent assaults, or blast injuries [1]. Incident estimates range from 108 to 332 hospitalised new cases per 100,000 population per year [2]. There are limited data on the incidence of TBI in low- and middle-income countries; however, epidemiological research from India, with an estimated population size of 1.2 billion, indicates nearly 2 million people sustain TBI each year [3].

TBI can result in long term or lifelong physical, cognitive, behavioural, and emotional consequences. As a result of these consequences, TBI is one of the most disabling injuries [4] and the leading cause of death and disability in children and adults from ages 1 to 44 [5]. The US Centre for Disease Control and Prevention estimates at least 3.17 million Americans, approximately 1.1% of the US population, are living with long-term disability as a result of TBI [6]. Even mild TBI (mTBI), which accounts for 80% to 90% of all TBIs, can cause long-term cognitive problems that may affect a person’s ability to perform daily activities and to return to work [7]. Given the incidence and severity of the condition, TBI poses a significant financial burden to society [8]. The lifetime cost per case of severe TBI is estimated at $396,331 USD, with disability and lost productivity costs outweighing medical and rehabilitation costs by a factor of 4 to 1 ($330,827 / $65,504) [9].

Worldwide, much research is conducted relevant to TBI and with the potential to improve outcomes for people with TBI [10]. However, translation of knowledge from research into practice takes considerable time and effort [11,12]. Concerted action is needed to facilitate this process, involving individuals, teams, organisations, and systems [13,14]. Knowledge translation (KT) is a way to close evidence-practice gaps, and has been defined as ‘a dynamic and iterative process that includes the synthesis, dissemination, exchange and ethically sound application of knowledge to improve health, provide more effective health services and products and strengthen the healthcare system’ [15].

The care of people with TBI includes many disciplines because patients often have a long journey of care through pre-hospital, hospital, rehabilitation, and community settings [16-18]. At the same time, these are relatively discrete professional communities, which provide opportunities for researchers and ‘research users’ to collaboratively shape research [19] and so ensure the research conducted is relevant to the TBI community and stakeholders.

**Figure 1:**
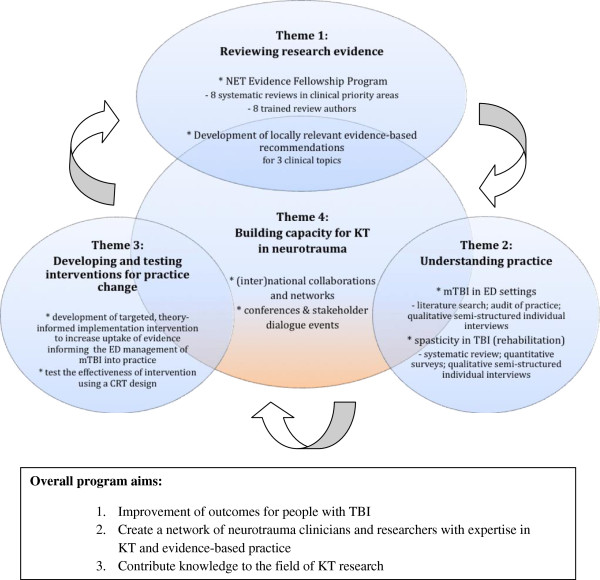
Overview of NET program themes, ‘high-level’ methods and overall program aims.

In November 2009, the Neurotrauma Evidence Translation (NET) program ( http://www.netprogram.org.au) commenced, funded by the Victorian Government’s Transport Accident Commission and Department of Innovation Industry and Regional Development, Australia. This five-year program provides opportunity to develop and sustain a coordinated collaborative approach to KT for TBI in Australia. The overall aims of the program are: to improve outcomes for people with TBI; to create a network of neurotrauma clinicians and researchers with expertise in KT and evidence-based practice; and to contribute knowledge to the field of KT research. The program includes a range of integrated activities in the knowledge-to-action-cycle [20]; captured in the following ‘themes’: reviewing research evidence; understanding practice; developing and testing interventions for practice change, and building capacity for KT in neurotrauma (see Figure 1).

This paper provides the rationale and an overview of the content of the NET program of KT research, including a brief description of the themes and the projects within each theme, their objectives, design, progress to date, and anticipated outcomes. Detailed methods and results of individual projects will be reported separately. At the time of writing of this manuscript, several components had already commenced.

## Methods

### Theme one: reviewing research evidence

For research evidence to be effectively implemented in clinical practice, it first needs to be synthesised and summarised and then made available in formats that are useful and relevant to the local setting [21]. Theme one activities are focused on reviewing the research evidence in TBI, generating knowledge products (*e.g.*, systematic reviews and their derivates, such as consensus statements, and quality indicators), and building capacity to conduct research synthesis. The theme builds on the work of the Global Evidence Mapping Initiative ( http://www.evidencemap.org) [22], which identified priority research questions relevant to TBI via key stakeholder involvement and produced evidence maps to illustrate the breadth and depth of available research addressing these questions. These evidence maps can be used to identify areas in need of synthesis (where evidence exists to guide practice) and ‘evidence gaps’ where primary research needs to be undertaken.

Theme one of the NET program has two main components: an Evidence Fellowship Program, and the development of locally relevant best practice recommendations.

### NET evidence fellowship program

Knowledge of current research evidence combined with clinical experience and patient preference is pivotal to evidence-based practice. To support evidence-based practice, clinicians need skills to search for, acquire, appraise, and interpret research findings. Barriers to evidence-based practice for clinicians have been well-documented, and include a lack of time, insufficient resources, and limited skills in accessing and applying research [23]. Fellowships offer clinicians protected time and provide access to resources and technical support that might otherwise be difficult to obtain [24,25].

The objectives of the NET Evidence Fellowship Program are to:

1. build skills and capacity for evidence-based practice within the neurotrauma community through mentoring clinicians to undertake systematic reviews;

2. encourage fellows to become future leaders and knowledge champions;

3. produce systematic reviews in priority neurotrauma topics to inform clinical practice.

To inform the design of the NET Evidence Fellowship Program, a systematic search was conducted in MEDLINE and EMBASE from 1996 to August 2011 to identify literature that described and evaluated evidence-based practice skill-development programs. Relevant papers were reviewed regarding their program concept, results, and experiences. The program was designed to incorporate the main findings from the systematic review and includes: recruitment of clinicians by targeting specific networks or recommendations by referral; access to training workshops (*e.g.*, Cochrane Collaboration author training); one-on-one mentoring and training by an experienced systematic reviewer and trainer with skills in searching, critical appraisal, interpretation of results and evidence-based decision making; and the provision of peer and administrative support.

Over the lifetime of the program, eight systematic reviews will be conducted predominantly on the effectiveness of interventions for the management of TBI. Five fellows have been recruited thus far and two protocols have been published [26,27].

### Developing locally relevant best practice recommendations

When evidence has been synthesised, and a reliable body of evidence exists, then efforts need to focus on converting the evidence into formats that are useful to end-users [21]. The second project of theme one focuses on developing best practice recommendations that are actionable, locally applicable, and consistent with best available research-based evidence. In our context, evidence synthesis needs to be suitable to underpin quality indicators and to define and measure best practice. Local adaptation of the evidence is essential [21], as potential dissimilarities in populations, interventions, or outcomes used in research [28,29], or organisational and cultural differences (*e.g.*, beliefs and values) between the research settings of the original studies and the site of implementation [30,31] may influence the relevance and feasibility of implementing a particular ‘body of evidence’ into a local setting.

Our process therefore encompasses the following steps: identifying current, high quality clinical practice guidelines (CPGs) and extracting recommendations; selecting strong recommendations in key clinical management areas; updating evidence and creating evidence overviews; discussing evidence and producing agreed ‘evidence statements’; discussing the relevance of the evidence with local stakeholders; and developing locally applicable actionable best practice recommendations, suitable for use as the basis of quality indicators. The process is reported in full elsewhere [32].

This project aims to develop recommendations in three areas of practice, relevant to TBI, in acute and rehabilitation phases of care. To date, the process has been completed for the management of mTBI in Australian emergency department (ED) settings [32,33].

### Theme two: understanding practice

Once evidence-based best practice has been agreed, current practice needs to be determined so as to identify gaps between evidence and practice and establish areas most in need of change [34]. In addition, current KT literature underlines the importance of researching and targeting the factors or determinants that may influence current practice and practice change prior to any efforts to change practice. These determinants may affect individual care providers, teams, organisations, or the wider healthcare system [35,36]. Interventions targeting prospectively identified determinants of change are more likely to improve professional practice than no intervention or dissemination of guidelines only [37]. A wide range of methods exist for the identification of these factors [37,38], including the use of theories of behavioural change [39,40]. The second theme focuses on understanding current practice and the factors that influence practice change within two clinical areas—management of mTBI in EDs and management of skeletal muscle spasticity following TBI in rehabilitation and community settings.

### Management of mTBI in ED settings

TBI is a frequent cause of presentation to EDs, and 70% to 90% of these are classified as mild [41,42]. The National Institutes of Health, USA, has declared that mTBI is a major public health problem and that effort to reduce disability after a mTBI should be a national research priority [43]. As the ED is the main, and often only, point of medical contact for these patients, ED care may have significant impact on the outcomes for these patients. Despite the existence of a variety of evidence-based CPGs on the management of mTBI, studies have shown considerable practice variation in a number of key clinical areas [44-47].

To explore current practice in the ED management of mTBI we triangulated data derived from the following methods: a literature search identifying previous studies on mTBI management in Australian EDs; an audit of management of mTBI in two Victorian EDs; and qualitative interviews with ED staff and directors in a purposeful sample of Victorian EDs to explore reported management. Our descriptions of current practices were compared with agreed local best practice, as determined in theme one [32,33], so as to estimate the presence and extent of any ‘knowledge-practice’ gaps.

Interviews with ED staff and directors also explored the individual, team, organisational, and system factors that may influence management and implementation of the agreed local best practice recommendations. We interviewed 42 participants (staff and directors) across 13 EDs until data saturation was reached [48]. Sites were selected to reflect both rural and metropolitan EDs. The design of the interview questions and analysis were guided by a theoretical framework [49]. Detailed methods and the findings of the interviews will inform the design of a targeted intervention (theme three) and will be reported in full in subsequent publications.

### Managing skeletal muscle spasticity following TBI in rehabilitation and community settings

Skeletal muscle spasticity is a major physical complication resulting from TBI. There is limited epidemiological data regarding the prevalence of spasticity following TBI, however it has been reported to affect more than one in ten patients with severe TBI [50]. Spasticity and its management have been identified as a priority topic by people with TBI and the multidisciplinary teams managing their care [22]. Interventions for managing skeletal muscle spasticity include pharmacological treatment (*e.g.*, baclofen, botulinum toxin A) and non-pharmacological interventions (*e.g.*, casting, stretching). Currently, little is known about the nature of current management of spasticity following TBI in rehabilitation or community settings.

We plan to explore current practice in the management of spasticity through conduct of a survey of Australian medical and allied health practitioners who manage spasticity following TBI. A random sample of practitioners working in rehabilitation and community care settings will be invited to complete a survey eliciting information about their assessment and treatment of spasticity following TBI. Our estimates of current practice will be compared with systematic review evidence supporting the use of interventions for managing skeletal muscle spasticity following TBI, as determined in theme one [26], so enabling us to estimate the size of the knowledge-practice gap. In-depth interviews with a purposeful sample of medical and allied health practitioners will then be conducted to explore the factors influencing current practice and the determinants of practice change (*i.e.*, the factors that influence the actual change process).

### Theme three: developing and testing interventions for practice change

Once the factors influencing practice and practice change have been identified, the next steps are to develop a KT intervention aiming to address these factors, and to test its effectiveness [51]. We aim to use a theory-informed approach to intervention development [52]. The use of theory can offer a generalisable framework for considering effectiveness across different clinical conditions and settings [53]. Given the wide variety of factors that may influence practice change, designing interventions using multiple theoretical perspectives minimises the chance of overlooking important factors [39] and is more likely to promote understanding about how and why change occurs. Research in this theme will build on work we have completed on the development of complex implementation interventions in other settings [52,54,55] and be informed by themes one and two.

Theme three aims to improve outcomes for patients with mTBI presenting to the ED through implementation of the locally relevant best practice recommendations developed in theme one. More specifically, the objectives are:

1. to systematically develop a targeted, theory-informed, and evidence-based implementation intervention to increase uptake of evidence informing the ED management of mTBI into practice;

2. to test in a cluster randomised trial (CRT) the effectiveness of this intervention in changing practice compared with passive dissemination of the recommendations;

3. to conduct a process evaluation alongside the CRT to understand the pathway of change.

A systematic process to map intervention components to identified determinants of practice change will be used in developing the targeted theory-informed intervention [51,52,56].

We will conduct a CRT to test whether the intervention is effective in improving the uptake of key evidence-based recommendations in the management of mTBI. Hospital EDs will form the clusters. A randomised design is the preferred design to evaluate the effectiveness of an intervention because it minimises bias in estimating intervention effects [57,58]. In this study, clusters (hospital EDs) have been chosen because the intervention is targeted at the team of ED staff, and EDs represent patient populations in geographical areas, precluding the use of an individually randomised design [59,60]. Intervention sites will receive the targeted, theory-informed intervention, while control sites will receive passive dissemination of the recommendations. A process evaluation will be completed to measure factors along the causal pathway of change and understand why change has or has not taken place [61]. Detailed methods of the CRT will be published as a study protocol.

### Theme four: building capacity for KT in neurotrauma

The overarching and supporting theme of the NET program is to build sustained capacity and infrastructure for KT in TBI [62], thereby improving patient outcomes as well as contributing to the science of KT research. To stimulate widespread and sustained uptake of research results into clinical practice beyond those forming the NET program, we aim to contribute to building a culture of research-informed decision making.

Building capacity and developing infrastructure requires investment in resources and structures, such as innovative training, skill development and support for practitioners, and sustained commitment from clinical leaders. Cross sector relationships and partnerships are needed between researchers, clinicians, policy makers, and healthcare consumers to build forums for exchange. To inform this, we need evaluation of what works to implement research into practice; for whom, why, and at what cost. Theme four will harness activities and networks from themes one to three to establish resources and systems, develop a workforce with KT skills, and foster a sustainable neurotrauma KT structure and culture. Specific functions under theme four include the coordination and hosting of three KT conferences over the course of the program, stakeholder dialogue events, and brokerage of relationships across disciplines and sectors through advisory structures and shared prioritisation.

The NET program has a program-wide multidisciplinary steering committee, which provides strategic direction to the program, offers practical support towards achieving the overarching aims of the program, and fosters links between the NET program and its identified stakeholder groups—the neurotrauma clinical community, consumers, policy makers, and funders. A number of networks and collaborations have been formed spanning Australia, Canada, USA, and UK, and these teams are exploring new neurotrauma KT initiatives that build upon the activities in the NET program.

## Conclusion

The NET program is a coordinated approach to KT in TBI in Australia. The program comprises a series of interlinked projects spanning varying clinical environments and disciplines, anchored within four themes representing core KT activities: reviewing research evidence; understanding practice; developing and testing interventions for practice change, and building capacity for KT in neurotrauma. The program aims to contribute to improving care for patients with TBI in Australia, and to contribute to the science of KT research.

## Abbreviations

CPG: Clinical Practice Guideline; CRT: Cluster Randomised Trial; ED: Emergency Department; (m)TBI: (mild) Traumatic Brain Injury; NET: Neurotrauma Evidence Translation; KT: Knowledge Translation.

## Competing interests

Denise O’Connor is an Associate Editor for Implementation Science. The authors declare they have no other competing interests.

## Authors’ contributions

Sally Green and Russell Gruen conceived of the program and wrote the original grant proposal. Joanne McKenzie and Denise O’Connor contributed to sections of the original grant proposal. Marije Bosch wrote the first draft of the manuscript and prepared the revised versions. All other authors critically reviewed and contributed to draft revisions, and read and approved the final version of the manuscript.
